# Anthropogenic Impacts on Aquatic Insects in Six Streams of South Western Ghats

**DOI:** 10.1673/031.007.3701

**Published:** 2007-06-04

**Authors:** S. Dinakaran, S. Anbalagan

**Affiliations:** Centre for Research in Aquatic Entomology, The Madura College, Madurai - 625 011. Tamil Nadu, India

**Keywords:** Human impacts, stream insects, water quality

## Abstract

Diversity patterns of aquatic insects among sampling sites lying with!ç the unprotected and protected areas of Western Ghats were studied. This study primarily emphasizes whether anthropogenic influence is the prime cause for the presence of aquatic insects especialIy of pollution-sensitive organisms belonging to the orders Ephemeroptera, Plecoptera and Trichoptera, or to factors such as the physico-chemical features of the water, or sampling methods. Six streams were sampled quantitatively, of which three streams (Abbifalls, Monkey falls and SiIver Cascade) were within protected areas and the remaining three streams (Kumbakarai, Shenbagadevi and Manimutharu falls) were in unprotected areas. A total of 3,209 individual aquatic insects belonging to 25 genera, 18 families and 7 orders were collected. The highest species richness and abundance was observed in Monkey falls followed by Kumbakkarai falls. Large çumbers of more habitat-sensitive organisms such as *Ecdyonurus sp., Epeorus sp., Thalerosphyrus* sp., *Euthraulus sp.,* and *Nathanella sp.,* were found in Monkey falls. Though the species assemblage was somewhat different, pollution-sensitive taxa were also observed in Kumbakkarai falls. Shenbagadevi and Manimutharu falls had a lower diversity of aquatic insects. The likely causes of these differences are discussed.

## Introduction

**Figure 1.**
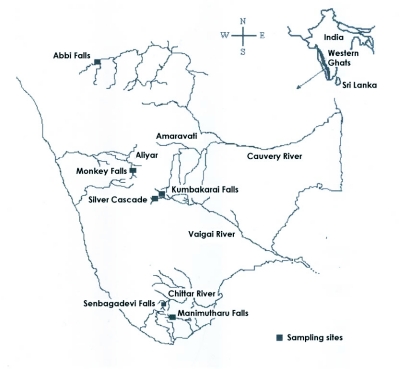
Map showing location of sampling sites.

The biological approach for assessing streams and rivers is the use of benthic macroinvertebrates, especially aquatic insects, as indicators of pollution (Dudgeon 1999). There is a high probability that human-induced change will also result in a change in the composition of the benthic community. The use of benthic macroinvertebrates is widespread and constitutes the basis for most aquatic biomonitoring programs currently in use (Metcalfe 1989; Rosenberg & Resh 1993). In particular, the influence of humans on streams and rivers used for recreational purposes, such as community bathing for personal hygiene, has caused habitat impairment in several areas lying within the state of Tamil Nadu. The objectives of the present study were to establish the faunal changes that have been occurred in popular tourist places such as Abbifalls, Monkey falls, Silver Cascade, Kumbakarai falls, Shenbagadevi falls and Manimutharu falls. Abbifalls, Monkey falls and Silver Cascade are within protected areas, while Kumbakarai falls, Shenbagadevi falls and Manimutharu falls are in unprotected areas. This is an effort to highlight seasonal, hydrobiological and physico-chemical changes that have been recorded against the existence of aquatic insects, and to analyze the diversity patterns existing among the sampling sites lying within the unprotected and protected areas. We hoped to determine whether anthropogenic influence is the prime cause for the presence of certain aquatic insects, or factors such as physico-chemical profiles or sampling methods are more likely causative factors.

## Materials and Methods

During a pilot field trip survey of the six streams, several different habitat types were identified while walking along the streamside along the entire stretch. The ranges of microhabitats were noted. Inside the stream aquatic insects were surveyed on cobbles, rock, large woody debris, decaying leaves and dislodgable boulders. Habitat heterogeneity was expressed in terms of percentage of boulders and cobbles, canopy cover and shade. The taxa of riparian vegetation, and stream bank stability and channelisation details were noted. The percentages of total tree cover, and tree shade (overhanging trees) were determined using densiometer readings. The rationale of selection of specific sampling sites in the present survey is that they should reflect the reference conditions, as opposed to impacted conditions enabling collection of baseline data for future biomonitoring studies. For sampling, streams from four different basins viz, Moyar basin, Azhiyaru basin, Vaigai and Tamarabharani basin were selected. Six sampling stations that are important touist spots were chosen for the present investigation. Among these, Abbi falls lies within Moyar basin, Monkey falls lies in Azhiyaru basin, Kumbakkarai and Silvercascade lies in Vaigai basin, and the Manimutharu and Shenbagadevi falls lie within Tamarabharani basin (Figure 1). Streams were sampled in 2004 in three different monsoons: Pre - monsoon (October), Northeast monsoon (November), and Post monsoon (December).

**Table 1.**
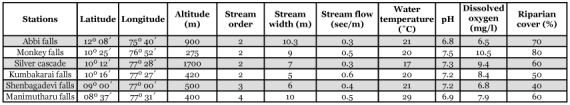
Mean value of physico - chemical characteristics of six different streams during Pre- monsoon, Northeast monsoon and Post monsoon 2004.

## Aquatic insects sampling

A total of six sampling sations were selected and three replicates in each station were sampled for this investigation. Physico-chemical variables were measured based on the procedures suggested in APHA (1995). Individual site description and physico-chemical features are given in Table 1. Aquatic insects were collected in a kick-net, running from the edge to the middle of each sampling station, with a 5 min kick sampling time. The kick-net was plastic and had a mesh size of 0.5 mm. Each habitat (e.g., riffle, pool, edge) was sampled proportionally to its representation at the site (Burton and Sivaramakrishnan 1993). The organisms were then removed from the net surface and were preserved immediately in 70% ethyl alcohol. All specimens from each of the six sampling stations were sorted and identified

**Table 2.**
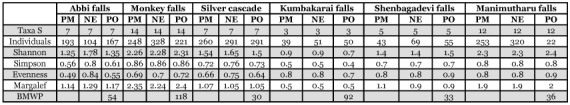
Diversity indices of aquatic insects in the six sampling stations during Pre - monsoon (PM), Northeast monsoon (NE) and Post monsoon (PM).

## Data analysis

In each sampling station the Shannon-Wiener diversity Index, Simpson's diversity index, Pielou evenness index and Margalef richness index were estimated. Calculations were done using package PAST version 1.42. The correspondence analysis (CA) was calculated to compare the faunal structure (CA= In (*x*+1) - transformed abundance distribution of taxa) of sampling sites and relay plots were ordered according to CA column scores. It was used to show the taxa ordered according to their positions along the gradients, and for each taxon the corresponding plot should ideally show a unimodel peak, partly overlapping with the peak of the next taxon along the gradient. Each data point is plotted with CA first axis row scores on the horizontal axis (Hennebert and Lees 1991). Principal component analysis was applied to relate the relationship between faunal changes and physico-chemical variables, and the biological monitoring working party index (BMWP) was used to evaluate the biotic integrity of communities. BMWP analysis was based on Armitageet al. 1983.

**Figure 2.**
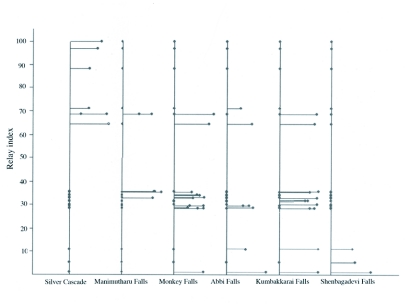
Relay plot of sampling locations by correspondence analysis. An illustration of the within-station variability of the faunal structure.

**Figure 3.**
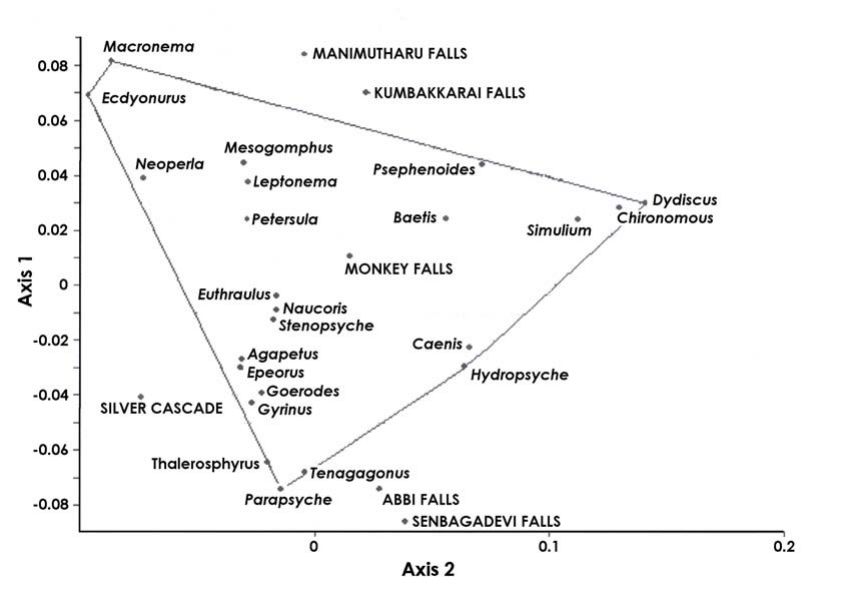
Ordination of sampling locations by correspondence analysis.

**Figure 4:**
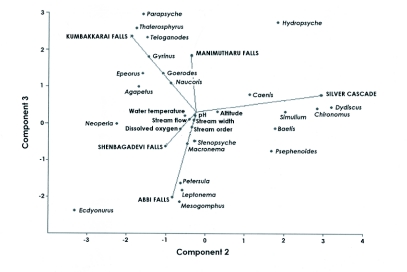
Biplot showed the abundance of taxa with physico-chemical parameters in six sampling sites by principal component analysis.

## Results

Physico-chemical parameters of sampling sites are listed in Tablet A total of 3209 individuals of aquatic insects belonging to 25 genera, 18 families and 7 orders were collected. Diversity indecies (Shannon-Weiner index, Simpson's index), richness (Margalef index) and evenness (Pielou index) for three different seasons were calculated (Table 2). Shannon-Weiner and Simpson's index were higher in Monkey falls and lower in Kumabakkarai falls. Species richness (Margalef) was higher in Monkey falls and lower in Shenbagadevi falls. Values on evenness index showed little contrast; it was highest in monsoon and lowest in pre - monsoon. The Margalef index was highest during post monsoon and lowest during monsoon (Table2).

Correspondence analysis clearly illustrated the change of community structure along with longitudinal gradient. Samples were clustered with the relay index from Abbi falls to Kumbakkarai falls and there was a gradual shift from Silver cascade to Shenbagadevi falls (Figure 3). This gradient might be as a result of a gradual shift between Trichoptera and Ephemeroptera *(Macronema* sp.in Manimutharu falls; *Ecdyonurus* sp. in Kumbakkarai falls) to Coleoptera and Odonata in Monkey falls (Figure 3). Faunal variations along F1 axis seemed to be essentially related to stream flowing characteristics (i.e. more or less water) of sites. Assemblage of *Wormaldia, Stenopsyche, Hydropsyche, Agapteus, Epeorus, Goerodes, Thalerosphyrus, Tenagogonus* and *Parapsyche* in the sampling sites of Shenbagadevi falls, Silver cascade and Abbi falls on the negative F1 axis revealed that they are the representatives of high stream flowing characteristics rather than with the sites of Manimutharu falls, Kumbakkarai falls and Monkey falls. Assemblages of aquatic insects on these three sites were the representatives of low flow stream characteristics. Principal component analysis on stream flow, stream width and water temperature showed significant relationships with species abundance (Figure 4).

BMWP score and taxa richness were displayed for the six sampling sites (Figure 4) so as to highlight the relationship existing among them. Higher taxonomic richness and BMWP score in Monkey falls followed by Kumbakkarai fall s suggested the pristine nature of the stream since they were least exposed to anthropogenic impact when compared to Manimutharu falls, Silver cascade and Shenbagadevi falls (Table2). Moderate BMWP values and taxonomic richness in Abbi falls might be due to other physico-chemical features in particular, stream characteristics rather than with anthropogenic impact. Similarity of the faunal composition of the six streams was very distinct (Figure 6).

**Figure 5.**
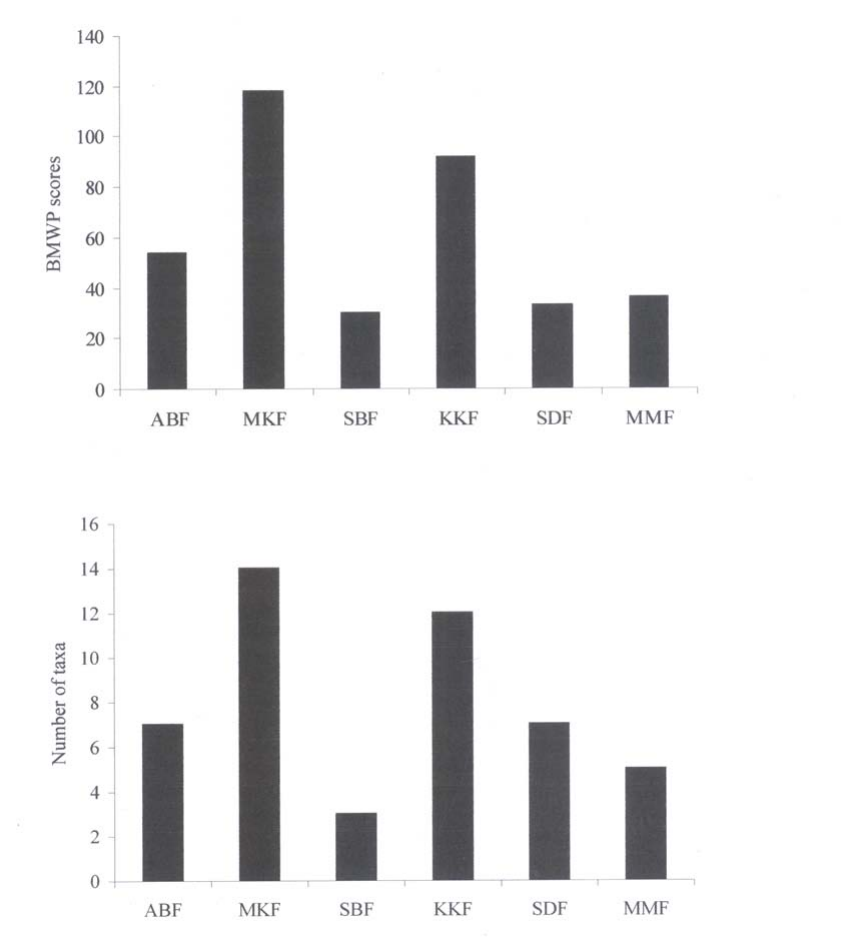
Biological Monitoring Working Party (BMWP) index scores and taxonomic richness of six streams (ABF- Abbi falls; MKF- Monkey falls; SDF- Shenbagadevi falls; SBF- Silver cascade; MMF- Manimutharu falls and KKF- Kumbakkarai falls).

## Discussion

Species diversity patterns in selected streams of Western Ghats have been well studied (Anbalagan and Dinakaran 2006; Dinakaran and Anbalagan 2006; Subramanian and Sivaramakrishnan 2005; Anbalagan et al. 2004). In the present study aquatic habitats in six different streams were examined. Existing concepts that have been developed to predict biodiversity along a river (Chown and Gaston 2000; Willig 2001) were supported by these data. Each stream exhibited a distinct latitudinal pattern sequence in species diversity, which emphasizes the uniqueness of these streams. The major aquatic insect taxa of Ephemeroptera, Plecoptera, and Trichoptera complexes are consequently absent in the Tampiraprani river (Martin et al. 2000). In contrast, the present study found abundance and species richness of Ephemeroptera, Plecoptera, and Trichoptera complexes in the Tampiraparani river basin of Shenbagadevi falls. Hynes (1975) already proposed that ‘every stream is likely to be individual’, moreover, each substrate type exhibits a very distinct community, and faunal similarity. The “individuality” of streams as well as of substrate types, however, has been challenged by anthropogenic impacts. It not only eliminates the lateral habitats but also leads to a homogenization of aquatic communities. Along the unprotected areas, for example, main stem habitats were species-poor. This might be due to inadequate sampling strategies adopted for this study as species diversity would be higher if the studies were conducted across the substrate types found in tributaries. Tributaries are the least affected segment by human beings.

**Figure 6.**
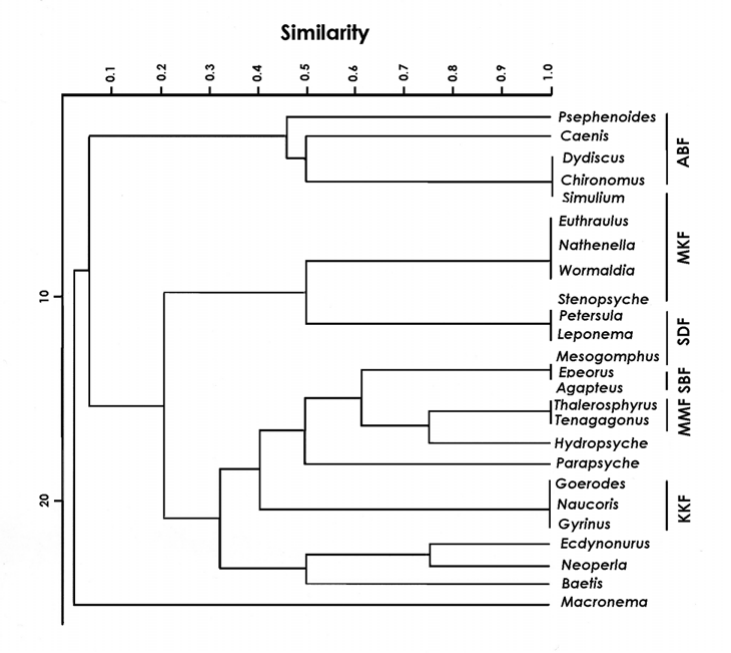
Dendogram showing similarity of faunal composition between six streams (ABF-Abbi falls; MKF-Monkey falls; SDF- Shenbagadevi falls; SBF- Silver cascade; MMF- Manimutharu falls and KKF- Kumbakkarai falls).

Tributaries, on the other hand, not only differ from the main channel with respect to environmental properties, but also are assumed to enhance the local heterogeneity at the confluence with the main channel (Benda et al. 2004). Brown & Coon (1994) reported higher fish density and different community composition in tributaries when compared to the channel (lower Missouri river, USA). They found a gradient in the faunal assemblage from small tributaries to large river sections, which corresponded to an environmental gradient from shallow streams with coarse substrate to deep rivers with finer sediments. The potential importance of tributaries for main stem communities is virtually unexplored. Tributaries may serve as important refugia for recolonising the main channel after disturbances (floods, droughts, pollution), and they are important habitats for early life stages of fish and invertebrates (Bruns et al. 1984) Rice et al. 2001).

Even if the concept of –river health– (often seen as being analogous with –human health– when applied to the evaluation of river condition - Resh et al. 1995) remains subject to considerable debate (for example, Steedman 1994; Meyer 1997; Bunn et al. 1999; Karr 1999; Morris and Thorns 1999), it is clear that the assessment of river health involves comparisons (Morris and Thorns 1999). In this way, appropriate metrics of river health, including measures of structure and functions of biotic and physico-chemical components, may be compared between sites keeping in mind that conditions affecting the ecological health of rivers (i.e. biogeographic processes in the regional climatic and geological context) vary geographically. Applying this approach to the sampling sites suggested that the protected areas along the Tamil Nadu area might be considered to be a healthy river, exhibiting both high biodiversity and ‘reasonably good’ water quality. The region between the Abbi and Kumbakkarai falls and the Silver cascade and Shebagadevi falls showed the influence of a high degree of human impact on stream integrity. This sector was influenced by discharge of domestic effluents and by community bathing for personal hygiene. Indeed, channelization might have changed the characteristics of invertebrate habitats through channel straightening and eradication of pool-riffle sequences, together with a reduction of the substrate mosaic heterogeneity (Boon 1988). In addition, embankment construction has led to an artificial channel reducing the availability of littoral refugia that lessen the impact of both natural (e.g. unpredictable patterns of discharge) and anthropogenic (bathing) disturbances on the biota (Townsend 1989; Townsend and Riley 1999). This combination of factors contributed to a drastic decrease in biotic indices corresponding to a dramatic reduction of pollution intolerant taxa in macrobenthic assemblages. Thus, taxonomic richness was lower in the extensively straightened, deepened and embanked sampling sector, but increased in the non-channelized stream section (Monkey falls) situated in a protected area, where large numbers of more habitat-sensitive organisms (Ephemeroptera, Plecoptera, and Trichoptera complexes.) were found. The reason for the rich biodiversity of Monkey falls would be due to the pristine condition of the stream, since the sampling site lies within the protected area. In this way, although there was a drastic, sequential unprotected area of benthic assemblages in the southern Western Ghats, rare specimens of habitat-sensitive organisms such as Ephemeroptera Plecoptera and Trichoptera still occurred in the unprotected areas and total resurgence of pollution sensitive taxa in unprotected or polluted sites would be possible if the riparian corridor is protected.

## Abbreviations

BMWP: biological monitoring working party index
